# Protocol for a systematic review of the clinical effectiveness of pre-hospital blood components compared to other resuscitative fluids in patients with major traumatic haemorrhage

**DOI:** 10.1186/2046-4053-3-123

**Published:** 2014-10-24

**Authors:** Janine Dretzke, Iain M Smith, Robert H James, Mark J Midwinter

**Affiliations:** 1 Department of Public Health, Epidemiology and Biostatistics, School of Health and Population Sciences, College of Medical & Dental Sciences, University of Birmingham, Birmingham B15 2TT, UK; 2 NIHR Surgical Reconstruction & Microbiology Research Centre, Queen Elizabeth Hospital Birmingham, Edgbaston, Birmingham B15 2TH, UK; 3 Academic Department of Military Emergency Medicine, Royal Centre for Defence Medicine, Queen Elizabeth Hospital, Edgbaston, Birmingham B15 2TH, UK; 4 Academic Department of Military Surgery & Trauma, Royal Centre for Defence Medicine, Queen Elizabeth Hospital, Edgbaston, Birmingham B15 2TH, UK

**Keywords:** Pre-hospital, Resuscitation, Red blood cells, Plasma, Haemorrhage, Trauma, Military, Civilian, Systematic review

## Abstract

**Background:**

There is growing interest in the use of blood components for pre-hospital resuscitation of patients with major traumatic haemorrhage. It has been speculated that early resuscitation with blood components may have benefits in terms of treating trauma-induced coagulopathy, which in turn may influence survival. The proposed systematic review will evaluate the evidence on the clinical effectiveness of pre-hospital blood components (red blood cells and/or plasma or whole blood), in both civilian and military settings, compared with other resuscitation strategies in patients with major traumatic haemorrhage.

**Methods/design:**

Standard systematic review methods aimed at minimising bias will be employed for study identification, selection and data extraction. General medical and specialist databases will be searched; the search strategy will combine terms for the population, intervention and setting. Studies will be selected for review if the population includes adult patients with major traumatic haemorrhage who receive blood components in a pre-hospital setting (civilian or military). Systematic reviews, randomised and non-randomised controlled trials and controlled observational studies will be included. Uncontrolled studies will be considered depending on the volume of controlled evidence. Quality assessment will be tailored to different study designs. Both patient related and surrogate outcomes will be considered. Synthesis is likely to be primarily narrative, but meta-analyses and subgroup analyses will be undertaken where clinical and methodological homogeneity exists.

**Discussion:**

Given the increasing use by emergency services of blood components for pre-hospital resuscitation, this is a timely systematic review, which will attempt to clarify the evidence base for this practice. As far as the authors are aware, the proposed systematic review will be the first to address this topic.

**Systematic review registration:**

PROSPERO CRD42014013794

## Background

The in-hospital resuscitation of patients suffering from major traumatic haemorrhage has undergone a revolution over recent years, with the previous mainstay of resuscitation—crystalloid—being replaced by blood component therapy. Observational evidence from both military and civilian practice suggests a survival benefit from resuscitation with high ratios of plasma to packed red blood cells (pRBC) (so-called “haemostatic resuscitation” (HR)) [1,2]. It has been suggested that early use of blood products rather than crystalloids may convey a survival advantage, perhaps due to improved oxygen-carrying capacity, more effective volume expansion and by lessening the coagulopathy of trauma [3,4].

The pre-hospital setting is more complex. A previous randomised controlled trial [5] demonstrated that pre-hospital crystalloid resuscitation increased mortality and morbidity in patients who had suffered penetrating trauma. It is believed that early aggressive volume administration may lead to “clot blow-off”, i.e. clots dislodged due to increased arterial pressure, and rebleeding. Consequently, pre-hospital resuscitation moved towards restricted fluid regimes.

Military experience has proven that it is feasible and practical to provide blood products for transfusion in the pre-hospital arena [6]. Anecdote and limited observational evidence from British military casualty retrieval missions suggest a potential survival benefit from pre-hospital HR [7,8] but are confounded by factors such as increasingly liberal in-hospital transfusion practices [9]. However, a large observational study of pre-hospital blood products failed to identify any reduction in mortality or coagulopathy [10]. This is consistent with a civilian in-hospital study which suggested that HR’s effect is dependent on surgical haemorrhage control [11]. Other confounders of military studies include incomplete data (due to the nature of the combat environment) and inability to follow up patients—particularly those of other nationalities—following discharge. The military population is younger, almost exclusively male and fitter, with minimal comorbidity compared to civilian trauma patients. Mechanisms of injury differ; over 95% of severe battlefield trauma results from explosive events or gunshot from military weapons [10]—mechanisms almost unknown in UK civilian practice and rare in other countries. Consequently, perceived benefits from military pre-hospital cannot be assumed to translate directly to civilian practice.

Nonetheless, an increasing number of civilian pre-hospital retrieval services are carrying pRBCs for trauma resuscitation, which has significant cost and logistical implications. Pilot studies [12-14] have demonstrated the feasibility of pre-hospital pRBC use with complete traceability and minimal wastage (0.0%–1.6%), with no early transfusion reactions recorded. Studies on the effectiveness of pre-hospital pRBC [3,15,16] or plasma [17] transfusion in a civilian population suggest a benefit; however, these findings are limited by factors including retrospective design, non-standardised care and insufficient statistical power. There are at least two relevant ongoing randomised controlled trials [18,19].

Despite the existence of a number of primary studies as outlined above, a scoping search identified no existing systematic reviews. A systematic review [20] on all aspects of the acute management of trauma from 2011 was limited to RCTs and did not find any relevant studies. US guidelines from 2009 [21] had a limited search strategy, and searches were performed in 2007; this report included two potentially relevant primary studies [22,23]. A narrative review from 2013 [24] with a limited search strategy identified only one relevant study [12].

Pre-hospital resuscitation with blood components has already been adopted as routine practice in some emergency services. Urgent clarification of the evidence base is required. The aim is therefore to undertake a systematic review of the evidence on the clinical effectiveness of pre-hospital blood components (red blood cells and/or plasma or whole blood), in both civilian and military settings, compared with other resuscitative fluids in patients with major traumatic haemorrhage.

## Methods/design

Standard systematic review methodology aimed at minimising bias will be employed, and reporting will follow the Preferred Reporting Items for Systematic Reviews and Meta-Analyses (PRISMA) guidelines [11]. This protocol is registered with PROSPERO (CRD42014013794).

### Searches

The following sources will be searched for primary studies:

● Bibliographic databases—MEDLINE, MEDLINE In Process and EMBASE via Ovid and The Cochrane Library (CENTRAL databases)

● UK Blood Services Transfusion Evidence Library

● Defence Medical Library Service (Ministry of Defence)

● Science Citation Index (ISI) for citation searching

● Current controlled trials (http://www.controlled-trials.com/), WHO International Clinical Trials Registry Platform (ICTRP) and ClinicalTrials.gov for ongoing studies

● Specialist abstract and conference proceeding resources (British Library’s ZETOC and ISI Proceedings)

● Checking of citation lists of included studies and relevant reviews

● Contact with study authors and researchers of ongoing trials

● Contact with experts in the field (e.g. THOR—The Trauma Hemostasis and Oxygenation Research Network, the Cochrane Pre-hospital and Emergency Care group)

● Hand searching may be performed for relevant conference abstract books

A combination of alternative text and MeSH terms relating to the condition (haemorrhage), intervention (blood components) and setting (pre-hospital) will be utilised.

There will be no language restrictions applied to the searches. Given that the number of relevant primary studies is likely to be small, and the study design (terminology) variable, searches will be broad and study design filters will not be used. There will also be no restriction of searches by comparators or outcomes. A sample search strategy for MEDLINE is provided in Additional file 1.

Search results will be entered into electronic databases (EndNote version X7.1, Thomson Reuters, New York) to facilitate record keeping, duplicate removal, study selection and document writing. Titles (and abstracts where available) of articles identified by the searches will be screened by two reviewers, independently, for relevance to the review question using pre-specified screening criteria. This process will be aimed at removing non-relevant studies. Full text copies of potentially relevant articles will be acquired and assessed independently against the inclusion criteria by two reviewers. Discrepancy between reviewers will be resolved by discussion or by referring to a third reviewer. Where necessary, translation (full/part) of non-English language articles will be undertaken to facilitate this process and subsequent reviewing; the review team has access to a wide range of translators. The study selection process will be illustrated using a PRISMA flow diagram. Reference management software will be used to record reviewer decisions, including reasons for exclusion.

### Selection criteria

#### Study design

Based on scoping searches, relevant studies are most likely to be (retrospective) cohorts or uncontrolled studies (case series). Inclusion of studies will be limited in the first instance to controlled studies (randomised or non-randomised, prospective or retrospective, concomitant or historical control). It is likely that evidence from controlled studies will be limited (≤10 studies); if this is the case, inclusion will be extended to uncontrolled studies.

#### Patient group

The patient group will be individuals aged ≥16 with major traumatic haemorrhage with hypotension (as defined by study authors). There will be no restriction on type, mechanism or location of injury, or co-existing conditions.

#### Intervention

Any blood components likely to be administered in a pre-hospital setting, including the following: whole blood, RBCs alone, RBCs and plasma (any ratio) or plasma alone.

#### Comparator (for controlled studies)

Any other resuscitative fluid (e.g. crystalloids, colloids) or any of the interventions compared against each other, with or without additional agents such as tranexamic acid.

#### Setting

The setting is pre-hospital, i.e. administration of fluids at the point of injury or in transit to hospital (ground or air transport). Both civilian and military environments will be eligible.

#### Outcomes

There will be no restriction by outcome. The primary outcome of interest is survival; however, studies are unlikely to be powered to show any differences. The following (secondary) outcomes will therefore also be considered:

● Lactate concentration

● Blood gases

● Measures of coagulopathy

● Additional blood components (or other fluids) given in hospital

● Organ failure

● Infection

● Adverse events associated with intervention

● Outcomes relating to the feasibility of using blood components in a pre-hospital setting (e.g. wastage and transfusion reactions)

### Data extraction

Data extraction will be conducted by one reviewer using a standardised, piloted data extraction form and checked by a second reviewer. Disagreements will be resolved through discussion or referral to a third reviewer. For each study, the data required on (but not limited to) the following will be sought:

#### Study characteristics

● Country of origin

● Study design

● Setting (civilian or military)

● Sample size

● Length of follow-up

#### Population

● Patient inclusion and exclusion criteria (including definition of hypotension)

● Location, mechanism and severity of injury (e.g. as defined by the Injury Severity Score or New Injury Severity Score)

#### Intervention/comparator

● Combination/ratio of blood components and quantity administered

● Comparator fluid and quantity administered

● Location of administration (e.g. at the scene or in transit)

● Characteristics/training of individual administering fluids

#### Results

● Completeness of follow-up

● Outcome measures

● Statistical methods employed (e.g. for adjusting for confounders)

● Findings

● Effect sizes and associated uncertainty

### Quality assessment

Data will be extracted to allow quality assessment of the included studies. Quality assessment will be based on tools specific to a given study design.

For randomised and non-randomised controlled trials (should any be identified), quality assessment will be based on the risk of bias tool from the Cochrane Handbook [25]. The Newcastle-Ottawa Scale (NOS) [26] will be used for cohort or case-control studies. Some of the confounding factors likely to arise in non-randomised studies include the following: severity and mechanism of injury, distance between site of injury and hospital, transportation time, co-interventions, characteristics of individual administering fluids and adherence to fluid protocols. When assessing study quality, the extent to which confounders have been given adequate consideration, both in reporting and analysis, will be examined.

For uncontrolled observational studies, details will be sought on population characteristics (including representativeness and eligibility criteria), intervention and adequacy of assessment of relevant outcomes and of follow-up (e.g. at least 30 days for survival (civilian population) or up to point of discharge to other healthcare service (military population)).

In addition to the methodological criteria listed above, the GRADE [27] framework may be used to consider inconsistency between studies, precision of results, likelihood of publication bias and applicability of results to population(s) of interest.

### Analysis

Narrative synthesis of evidence will be undertaken for all included studies. This will include a narrative description and tabulation of main results across studies and/or for specific outcomes. Visual representation of results in Forest plots without pooling may also be considered. Summary measures for the main outcome of interest, survival, may be in the form of relative risk or hazard ratio (adjusted or unadjusted). For other outcomes (e.g. lactate concentration), mean differences (adjusted or unadjusted) may be reported. Based on preliminary findings, it is unlikely that there will be sufficient (similar) studies to conduct meta-analysis; however, appropriate meta-analytic methods will be employed where possible to combine data from similar studies. Assessment of clinical and methodological heterogeneity will be used to determine whether a fixed or random effects model is the most appropriate, rather than relying on the tests of heterogeneity from a fixed effect model to make such a decision [28]. The *I*^2^ statistic (which gives the percentage of the total variability in the data due to between-study heterogeneity) and the tau-squared statistic (which gives an estimate of the between-study variance) will be reported where appropriate. Where studies have reported time-to-event analyses, meta-analysis using the extracted hazard ratios and their variances will be undertaken, if possible. Evidence from studies of different design will not be quantitatively combined, but presented separately. Any adjusted and unadjusted results will also be presented separately. Presentation of results in Forest plots without a pooled summary estimate will be considered where pooling is not feasible.

For each meta-analysis containing 10 or more studies, the likelihood of publication bias will be investigated through the construction of funnel plots and appropriate statistical tests for small-study effects (such as the Peters Test [29]); that is, the tendency for smaller studies to provide more positive findings. It is well recognised that, especially where heterogeneity exists, publication bias may be one of a number of reasons for any small-study effects identified.

### Subgroup analysis

Subgroup analysis may include examining studies separately depending on pre-hospital setting (military or civilian) or where there is clear clinical heterogeneity between studies (e.g. in intervention/comparator, patient characteristics etc.). The robustness of any meta-analysis conclusions to the inclusion/exclusion of low quality studies (i.e. those at most risk of bias) may be assessed if feasible.

## Discussion

Emergency services, both in the UK and internationally, are already increasingly incorporating the use of blood components into pre-hospital resuscitation protocols for major traumatic haemorrhage. As far as the authors are aware, however, there have been no attempts to formally review the evidence base, and a systematic review is therefore urgently required. Whilst some studies appear to show benefits of pre-hospital blood components, there are methodological issues associated with many studies, which are mainly of a retrospective observational design. A thorough evaluation of study methodology and risk of bias as part of the proposed systematic review will not only help to make an assessment of the robustness of findings but may also help to inform future study design. Further, an assessment of the extent of transferability of findings from military research to a civilian population will be of interest, both for this systematic review question and potentially for other areas of trauma medicine.

## Abbreviations

HR: haemostatic resuscitation; pRBC: packed red blood cells; RBC: red blood cells.

## Competing interests

The authors declare that they have no competing interests.

## Authors’ contributions

MM is the guarantor. JD and IS drafted the manuscript and developed the search and methodological strategy. MM, IS and RJ provided clinical expertise. All authors read, provided feedback and approved the final manuscript.

## Supplementary Material

Additional file 1 **Sample search strategy.** Sample search strategy to identify relevant primary studies in MEDLINE. Click here for file
