# Supplementary material for: Protocol for a systematic review of the clinical effectiveness of pre-hospital blood components compared to other resuscitative fluids in patients with major traumatic haemorrhage
Source: Syst Rev. 2014 Oct 24;3:123. doi: 10.1186/2046-4053-3-123 (PMC4224592; doi:10.1186/2046-4053-3-123)
Supplement: Additional file 1 — Sample search strategy. Sample search strategy to identify relevant primary studies in MEDLINE. [file 2046-4053-3-123-S1.pdf]

Additional file 1 – Sample search strategy for MEDLINE

MEDLINE strategy

Database: Ovid MEDLINE(R) In-Process & Other Non-Indexed Citations and Ovid MEDLINE(R) <1946 to Present>
Search Strategy:
----
1 (red blood cell$ or red cell$).mp. [mp=title, abstract, original title, name of substance word, subject heading word, keyword heading word, protocol supplementary concept word, rare disease supplementary concept word, unique identifier]
2 (RBC or pRBC or PRC or RCC).mp. [mp=title, abstract, original title, name of substance word, subject heading word, keyword heading word, protocol supplementary concept word, rare disease supplementary concept word, unique identifier]
3 (blood product$ or blood component$ or whole blood).mp. [mp=title, abstract, original title, name of substance word, subject heading word, keyword heading word, protocol supplementary concept word, rare disease supplementary concept word, unique identifier]
4 blood administration$.mp.
5 (blood adj3 resuscitat$ or plasma adj3 resuscitat$).mp. [mp=title, abstract, original title, name of substance word, subject heading word, keyword heading word, protocol supplementary concept word, rare disease supplementary concept word, unique identifier]
6 (freeze dried plasma or fresh frozen plasma or liquid plasma or thawed plasma or spray dried plasma or lyophili?ed plasma or FDP or FFP).mp. [mp=title, abstract, original title, name of substance word, subject heading word, keyword heading word, protocol supplementary concept word, rare disease supplementary concept word, unique identifier]
7 (hemostatic resuscitat$ or haemostatic resuscitat$ or damage control resuscitat$).mp. [mp=title, abstract, original title, name of substance word, subject heading word, keyword heading word, protocol supplementary concept word, rare disease supplementary concept word, unique identifier]
8 1 or 2 or 3 or 4 or 5 or 6 or 7
9 (pre-hospital or prehospital or pretrauma or pre-trauma).mp. [mp=title, abstract, original title, name of substance word, subject heading word, keyword heading word, protocol supplementary concept word, rare disease supplementary concept word, unique identifier]
10 point of injury or point of wound$.mp.
11 (on scene or en route or in transit or retrieval).mp. [mp=title, abstract, original title, name of substance word, subject heading word, keyword heading word, protocol supplementary concept word, rare disease supplementary concept word, unique identifier]
12 out of hospital.mp.
13 (air or helicopter$ or aviation or rotary wing).mp. [mp=title, abstract, original title, name of substance word, subject heading word, keyword heading word, protocol supplementary concept word, rare disease supplementary concept word, unique identifier]

14 paramedic$.mp. or exp Allied Health Personnel/ or exp emergency responders/ or exp physicians/

15 exp first aid/ or exp emergency treatment/ or exp accident, traffic/

16 (evacuation$ or battlefield$ or wartime or military or casualt$).mp. [mp=title, abstract, original title, name of substance word, subject heading word, keyword heading word, protocol supplementary concept word, rare disease supplementary concept word, unique identifier]

17 exp Military Personnel/

18 (advanced trauma life support or ATLS).mp. [mp=title, abstract, original title, name of substance word, subject heading word, keyword heading word, protocol supplementary concept word, rare disease supplementary concept word, unique identifier]

19 (basic trauma life support or BTLS).mp. [mp=title, abstract, original title, name of substance word, subject heading word, keyword heading word, protocol supplementary concept word, rare disease supplementary concept word, unique identifier]

20 9 or 10 or 11 or 12 or 14 or 15 or 16 or 17 or 18 or 19

21 trauma.mp. or exp "Wounds and Injuries"/

22 injur$.mp.

23 (haemorrhag$ or hemorrhag$).mp. [mp=title, abstract, original title, name of substance word, subject heading word, keyword heading word, protocol supplementary concept word, rare disease supplementary concept word, unique identifier]

24 exp Hemorrhage/ or bleed$.mp.

25 shock.mp. or exp Shock, Traumatic/ or exp Shock, Hemorrhagic/

26 hypovol$.mp. or exp Hypovolemia/

27 (blood adj2 loss).mp. [mp=title, abstract, original title, name of substance word, subject heading word, keyword heading word, protocol supplementary concept word, rare disease supplementary concept word, unique identifier]

28 low blood pressure.mp. or hypotensi$.mp. or exp hypotension/

29 21 or 22 or 23 or 24 or 25 or 26 or 27 or 28

30 8 and 20 and 29
